# Supplementary material for: Identification of Novel Glycans in the Mucus Layer of Shark and Skate Skin
Source: Int J Mol Sci. 2023 Sep 20;24(18):14331. doi: 10.3390/ijms241814331 (PMC10532229; doi:10.3390/ijms241814331)
Supplement: Supplementary file 1 [file ijms-24-14331-s001.zip › supplementary figures.pdf]

Supplementary files

Supplementary File S1 (Excel file, list of glycans derived from mucin layer of sharks and skates).

Supplemental Figure S1

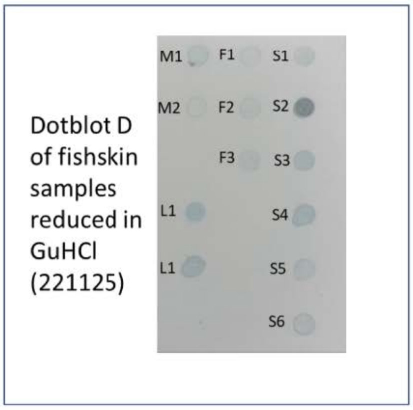

Supplementary Figure S1 (dot blot). Reduced and alkylated mucosal scrapings from spiny dogfish, chain catshark and little skate were dotblotted onto the pvdf membrane, followed by staining with Alcian Blue which stains for acidic glycans (see materials and methods). M1, M2: catshark (male), F1, F2, F3: catshark (female), S1-S6: spiny dogfish (female), L1, L2: Little skate (female). Although the relative abundance of acidic glycans in some of the samples was very low, the stain allowed for the identification of the location of the sample.

Supplementary Figure S2 PAS/AB staining

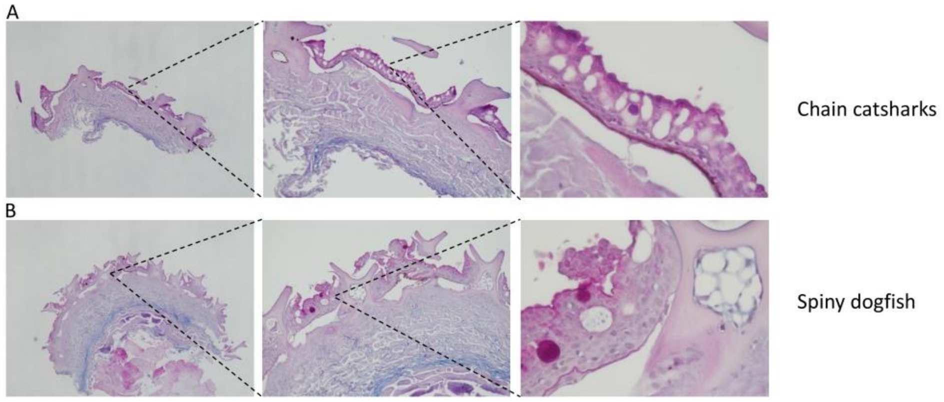

Supplementary Figure S2 (PAS/AB staining). All images are sagittal sections of skin biopsies. Representative images of one shark from each species are shown. PAS/AB staining of Chain catsharks (A) and spiny dogfish (B) are shown. Images were taken at 4x (500µm left image), 10x (middle image, 100µm), and 40x (right image 50µm). For each staining three or more individuals were imaged.

Supplementary Figure S3 Gram staining

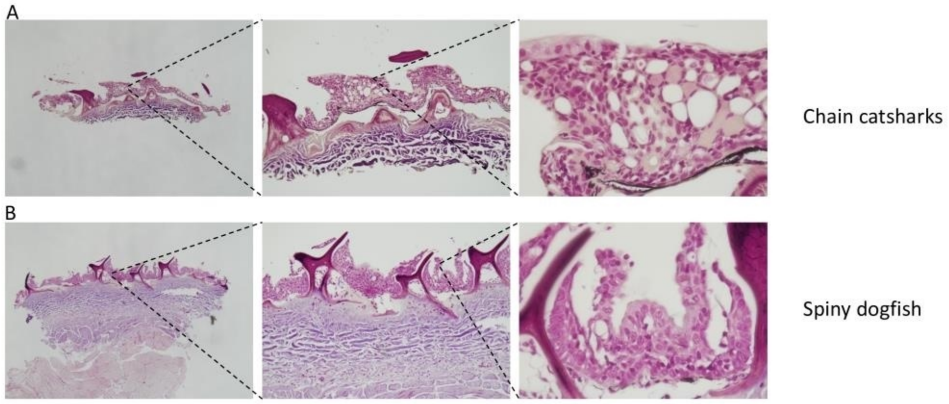

Supplementary Figure S3 (Gram staining). All images are sagittal sections of skin biopsies. Representative images of one shark from each species is shown. Gram staining of Chain catsharks (A) and spiny dogfish (B) is shown. Images were taken

at 4x (500μm left image), 10x (middle image, 100μm) and 40x (right image 50μm). For each staining, three or more individuals were imaged.
